# Potential gains in life expectancy by eliminating deaths from cardiovascular diseases and diabetes mellitus in the working life ages among Slovak population

**DOI:** 10.1186/s13561-018-0202-x

**Published:** 2018-08-22

**Authors:** Beata Gavurova, Tatiana Vagasova

**Affiliations:** Faculty of Economics, Technical University of Kosice, Nemcovej 32, 040 01 Kosice, Slovakia

**Keywords:** Life expectancy, Potential gains in life expectancy at birth, Working age groups, Mortality, Cardiovascular diseases, Diabetes mellitus

## Abstract

**Background:**

In recent years, high mortality from cardiovascular diseases (chronic ischemic heart disease, acute coronary syndrome, cerebrovascular diseases, atherosclerosis, hypertensive diseases) and diabetes mellitus have burdened economic and health system of the Slovak Republic considerably. By eliminating these deaths, the life expectancy could be prolonged. Since the mortality of population during working period has higher importance in terms of economic consequences of diseases, this article aims to assess the potential gains in life expectancy (PGLEs) of the Slovak population comparing the entire life span and working life-time.

**Methods:**

Data are obtained from the National Health Information Center mortality reports by sex during 1996–2014, and the method of constructing abridged life tables is used to compute the corresponding PGLEs. The added years, which would be gained by eliminating causes of deaths, are decomposed by the two sets of working age groups population (25–44 and 45–64 years).

**Results:**

The highest impact on life expectancy was recorded in chronic ischemic heart disease for both sexes aged 45–64 years (0.078 for males, 0.019 added years for females) over 1996–2014. However, they showed a small declining trend (− 16%) for males and even an increasing trend (2%) for females. At present, the labour force potential of working group (25–44 years) is most threatened by deaths from cerebrovascular diseases, while population of working age (45–64 years) by deaths from chronic ischemic heart disease. Relative importance of acute coronary syndrome for males (45–64 years) increased, when comparing the entire with working time life.

**Conclusions:**

The findings pose new and immediate challenges to policy makers and provoke discussion about prevention program strategies leading to increasing the life expectancy.

## Background

Cardiovascular diseases (CVD), as the main causes of death in developed countries, represent the most frequent causes of death in Slovakia. In particular, chronic ischemic heart disease, acute coronary syndrome, cerebrovascular diseases, atherosclerosis, hypertensive diseases. The number of deaths caused by CVD is estimated to 3.9 million per year in Europe, what accounts for 45% of all deaths in Europe [1]. The CVD mortality rates have been falling since 1970 in Western Europe [2], but in Eastern Europe remain comparatively high [3]. Specifically, CVD mortality rates decreased by 17–18% in Slovakia between 2001 and 2010, however, they decreased by 32–33% in France and by 31–34% in Germany during approximately the same time period [4].

Diabetes mellitus represents a very strong risk factor for CVD. The prevalence of diabetes mellitus for all age-groups worldwide was around 2.8% in 2000 and is estimated to be around 4.4% in 2030. The total number of people with diabetes is projected to rise from 171 million in 2000 to 366 million in 2030. The prevalence of diabetes is higher in men than women, however, in absolute terms there are more women with diabetes than men. The most important demographic change to diabetes prevalence across the world appears to be an increase in the proportion of people 65 years of age [5]. In 2012, there were 1.5 million of deaths due to diabetes mellitus and more than 2.2 million of deaths related to cardiovascular complications of diabetes mellitus. WHO predicts diabetes mellitus as 7th most common death by year 2030 [6]. In Slovakia we have around 400,000 patients with diabetes mellitus (7% of total population) and probably another 20–30% in pre-diabetes or with latent form of diabetes mellitus, and our predictions are around 15% by year 2030 [7]. The annual mortality rates from diabetes mellitus have been decreasing by 19.2% since 1990, an average of 0.8% a year [8].

In recent two decades, an overall decrease in mortality has contributed to the extension of life expectancy by 4 years in Slovakia, from 72.9 in 1996 to 76.9 years in 2014. It raises a question if it is a suitable growth of life expectancy, and how many additional years of life expectancy would be gained, if the main causes of deaths were eliminated. This issue can be measured by the potential gain in life expectancy (PGLE). The fall in mortality has changed according to a particular cause of death, sex, or age group of population. Therefore, an examination of the impact of the major causes of death on the life expectancy provides valuable information about the burden of these diseases on health system and economy of a particular country. Although, the high occurrence of morbidity and mortality in the group of retired people burdens the health system financing in a large extent, the mortality of population during working period has higher importance in terms of economic consequences of diseases. The high mortality among people of working age (the most 15–64 years) leads to the lower labour productivity as well as decelerating economic growth. Hence, the investigation of mortality in the working age groups may be a more relevant mortality indicator for a country compared with the group of retired people over age 65. In addition, the determination of underlying cause of deaths for older people can be difficult due to the high occurrence of comorbidities.

Costs of interventions related to CVD annually reach almost 200 billion EUR in the European Union. Diagnostics and treatment of cardiovascular diseases represent large economic burden on the health system in Slovakia. The European Union has set objectives with which the Slovak Republic identified and those contributed to better understanding of mechanisms supporting health, occurrence and development of diseases, improvements of possibilities of diagnostics, treatment and management of diseases leading to the healthy ageing [9]. Despite the advances in preventive cardiology, the cardiovascular mortality remains high. Metabolic syndrome is defined as simultaneous presence of lipid and non-lipid related cardiovascular and cardio-metabolic risk factors that significantly increase the risk of cardiovascular disorders, as well as diabetes type 2 and hypertension as compounds of metabolic syndrome [10].

There are several ways how to measure the burden of diseases on population [11, 12]. Previous studies [13–18] have explored the life tables containing the potential gains in life expectancy as a research method to determine the impact of diseases with the highest prevalence causing death on the life expectancy of population. However, most of these studies have focused on the overall groups of causes of death with relation to the demography but no with the specific implications on health interventions. For instance, Lai and Hardy [14] compared the PGLEs with the years of potential life lost by race and gender group from HIV, heart diseases, and cancer for US population. They found that better indicator for the measuring impact of disease deaths on a population is the PGLE because it is not influenced by the age and size population structure. In China, Liu et al. [15] proved that deaths from accidental injuries together with chronic diseases play a major role in influencing life expectancy. Conti et al. [13] found that AIDS and accidents have higher impact on life expectancy when considering working group (15–64 years), rather than 50% reduction in cardiovascular diseases deaths. Generally, it is necessary to observe a potential of the main causes of death and to encourage steps in the increase of life expectancy.

The aim of this paper is to analyse the influence of the deaths from CVD and diabetes mellitus on the life expectancy of the Slovak population comparing the entire life span and working life-time by sex from 1996 to 2014. These findings have a great potential for the creation of targeted prevention programs in Slovakia.

## Methods

Under the conditions of the contract, data on the number of deaths by five-year age groups for the period 1996–2014 were obtained from National Health Information Centre of Slovakia. Data on the mid-year population at the age groups in every year were downloaded from the Statistical Office of the Slovak Republic.

The PGLE reflects how many years on average a person would still live, if a given cause of death was eliminated. In other words, PGLE expresses years of life lost resulting from a certain disease in an age group. So, life expectancy could be extended for these years. The higher PGLE, the higher impact of the disease on life expectancy is.

For calculation of PGLEs it was needed to construct the life tables and the specific life tables regarding causes of deaths in the Slovak Republic in every year and an age group separately according to the methodical tutorials of Demographic Research Centre by Mészáros [19] and the National Vital Statistics Reports by Arias et al. [20]. We examined the life expectancy (e_x_) expressing the all causes of deaths and cause-eliminated life expectancy (${e}_x^{\left(-i\right)}$) by elimination of the certain causes of deaths. The Tenth Revision of the International Classification of Disease (ICD-10) was used to specify causes of death included in this analysis: diabetes mellitus (E10-E14), hypertensive diseases (I10-I13), acute coronary syndrome (I20-I22), chronic ischemic heart disease (I25), cerebrovascular diseases (I60–69), atherosclerosis, aortic aneurysm and dissection (I70-I72).

Cause-eliminated life expectancy (${e}_x^{\left(-i\right)}$) was the result from analysis of deaths caused by a certain disease and was calculated from the abridged life table minus causes of death.

According to the Mészáros [19], the first step is the calculation of the probabilities of survival (_n_*p*_*x*_) from the all-caused abridged life tables with the formula:

1 $$ {{}_n{}p}_x=1-{{}_n{}q}_x $$

where x – the exact age; n – the number of years in the age interval; _n_*q*_*x*_ - the probability of dying between the beginning of an age interval and before reaching the end of that age interval.

Then, the probabilities of death eliminating the i_th_ cause (${{}_n{}q}_x^{\left(-i\right)}$) were estimated by:

2 $$ {{}_n{}q}_x^{\left(-i\right)}=1-{{}_n{}p}_x^{\left(\frac{{{}_n{}D}_x-{{}_n{}D}_x^i}{{{}_n{}D}_x}\right)} $$

where ${{}_n{}D}_x$ - the number of deaths in the age interval *x* to *x + n* for all causes; ${{}_n{}D}_x^i$ - the number of deaths in the age interval x to x + n attributable to the i_th_ cause of death.

Arias et al. [20] report the number of person-years lived (${{}_n{}L}_x^{\left(-i\right)}$) in the age interval *x* to *x + n* was estimated for ages 0, 1, 5, 10,……, 95 by the formula:

3 $$ {{}_n{}L}_x^{\left(-i\right)}=\left(n-{{}_n{}f}_x\right)\bullet {l}_x^{\left(-i\right)}+{{}_n{}l}_x\bullet {l}_{x+n}^{\left(-i\right)} $$

where *n* = 1 for x = 0, *n* = 4 for x = 1, and *n* = 5 for x = 5, 10, ..., 95; ${{}_n{}l}_x$ – the number of persons from the original life table who survive to the beginning of each age interval; ${l}_x^{\left(-i\right)}$- the number of survivals from life table due to the i_th_ causes; *L*_*x*_ – the number of person-years from the original life table within an age interval *x* to *x + n*, and the quantities ${{}_n{}f}_x$ were estimated from the all-cause life table by:

4 $$ {{}_n{}f}_x=\frac{n\bullet \kern0.5em {{}_n{}l}_x-{{}_n{}L}_x}{l_x-{l}_{x+n}} $$

The last step is to calculate the number of person-years lived after exact age *x* (${T}_x^{\left(-i\right)}$) by:

5 $$ {T}_x^{\left(-i\right)}={L}_x^{\left(-i\right)}+{L}_{x+1}^{\left(-i\right)}+\dots +{L}_{95+}^{\left(-i\right)} $$

Finally, the cause-eliminated life expectancy (${e}_x^{\left(-i\right)}$) is calculated as:

6 $$ {e}_x^{\left(-i\right)}=\frac{T_x^{\left(-i\right)}}{l_x^{\left(-i\right)}} $$

Subsequently, the PGLE of a disease in a certain year is calculated as the difference between cause-eliminated life expectancy (${e}_x^{\left(-i\right)}$) and life expectancy (e_x_) in the same year.

7 $$ PGLE={e}_x^{\left(-i\right)}-{e}_x $$

The PGLEs for the working age group (the most 15–64 years) are those added years that would be gained, in the case of a particular cause of death elimination, before reaching the end of the working life span at age 65. In our analysis, we divided this general working group on two more particular age groups: young adults (25–44 years) and adults (45–64 years). It was carried out in compliance with the age classifications used for the topics of labour force participation and usage of health services recommended by United Nations [21].

To find out the PGLE in the working age groups of young adults (25–44 years) as well as adults (45–64 years), the partial life expectancies (e_25,44_ and e_45,64_) in the ages between 25 and 44 years as well as 45 and 64 years are calculated as:

8 $$ {e}_{25,44\ \left(45,64\right)}=\frac{T_{25\ (45)}-{T}_{44\ (64)}}{I_{25(45)}} $$

where T_25(45)_ – T_44(64)_ are the number of person-years lived in the age intervals 25–44 or 45–64, and I_25(45)_ are the number of survivors at 25 or 45 years of age in the life table. Similarly, the partial life expectancies in both age intervals after elimination of a particular cause of death are estimated. Finally, the PGLEs for the working life ages are expressed as the differences between cause-eliminated partial life expectancy and partial life expectancy for the working population aged 25–44 and 45–64 years separately.

## Results

### Trends of potential gains in life expectancy in the working age groups from 1996 to 2014

**Fig. 1:**
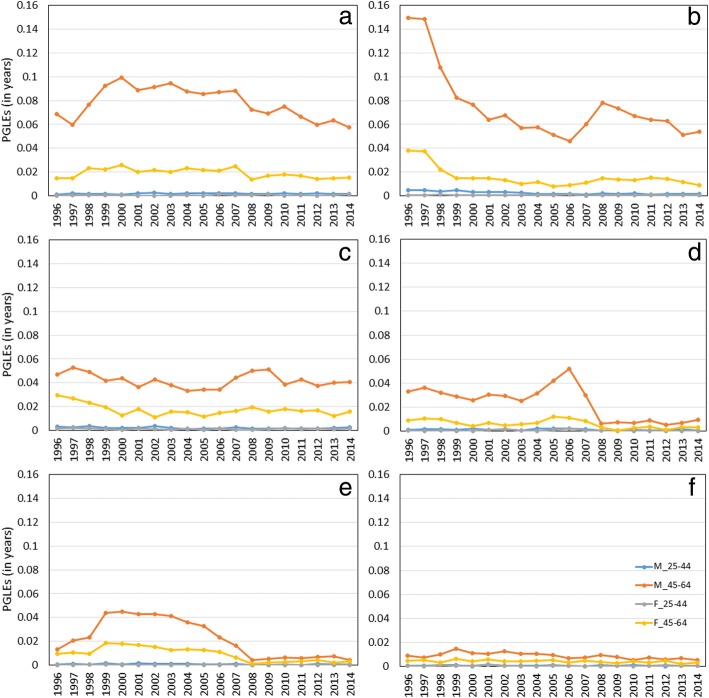
Trends of potential gains in life expectancy (PGLEs) by elimination of deaths from chronic ischemic heart disease (**a**), acute coronary syndrome (**b**), cerebrovascular diseases (**c**), atherosclerosis (**d**), hypertensive diseases (**e**), diabetes mellitus (**f**) for males (**M**) or females (**F**) Slovak population in the working age groups 25–44 or 45–64, 1996–2014

The PGLEs for the Slovak males and females in the working age groups 25–44 and 45–64 are plotted in Fig. 1. During the time span 1996–2014, the highest impacts of deaths from each of the examined diseases on life expectancy were recorded for males in the older age group (M_45–64), followed by females (F_45–64), young adult males (M_25–44), and young adult females (F_25–44). The PGLEs for young adults are approaching zero, that seems to be a negligible impact of deaths elimination on added years in life expectancy, even lower than 1 day added.

Considering men aged 45–64 years, the highest average PGLEs were recorded for chronic ischemic heart disease (0.078 years added), followed by acute coronary syndrome (0.075 years), cerebrovascular diseases (0.042 years), atherosclerosis (0.024 years), hypertensive diseases (0.022 years), and diabetes mellitus (0.009 years) from 1996 to 2014. For instance, after elimination of chronic ischemic heart disease deaths, a person at age 45 could be expected to live an average of 0.078 years longer before reaching the end of the working range at age 65. After the conversion potential gained years into days, 29 days of life could be added for men of older working age. We can see that the burdens of chronic ischemic heart disease and acute coronary syndrome are almost the same, while the burden of cerebrovascular diseases is lower by 45% on average. Considering women aged 45–64 years, the highest average PGLEs were also recorded for chronic ischemic heart disease (0.019 years, in other words 7 days added), cerebrovascular diseases (0.017 years), acute coronary syndrome (0.016 years), hypertensive diseases (0.009 years), atherosclerosis (0.006 years), and diabetes mellitus (0.004 years). For females, the PGLEs differences between the examined causes of death are not so remarkable than those for males.

Between 1996 and 2014, the PGLEs for males aged 45–64 years decreased for each of causes of deaths, similarly for females, except the small increase of chronic ischemic heart disease for females. Despite the fact that deaths from chronic ischemic heart disease had the highest impact on life expectancy, showed a small declining trend (− 16%) for males and even an increasing trend (2%) for females. The highest falls of PGLEs was recorded for atherosclerosis from 0.033 in 1996 to 0.01 in 2014 (− 71%), hypertensive diseases from 0.013 to 0.004 (− 68%), and acute coronary syndrome from 0.15 to 0.054 (− 64%) for males. The disease rank order is opposite for females aged 45–64 years, for acute coronary syndrome from 0.038 to 0.009 (− 77%), hypertensive diseases from 0.01 to 0.003 (− 69%), atherosclerosis from 0.009 to 0.003 (− 63%). As one could notice, the beginning of the economic crisis dated approximately from 2006 to 2008 might contribute to the rapid increase of deaths from acute coronary syndrome. On the contrary, the fast drop for atherosclerosis was showed in the same time span. As the above results show, the differences in PGLEs between the sexes still existed and men were more burdened of each disease than women. The highest gaps were found in acute coronary syndrome (4.8 times more for men) and chronic ischemic heart disease (4.1 times more for men), on the other hand, the lowest gap was resulted from diabetes mellitus (2.1 times more for men). The differences between the sex groups have been most reduced for atherosclerosis and hypertensive diseases since 2008.

### Potential gains in life expectancy in total expectation of life and in the working life ages

Table 1 shows the potential added years of life at birth, as well as of working life ages 25–44 and 45–64, through the elimination in mortality from six examined causes of death according to the sex. The PGLE is presented as an average value for the past 3 years 2012–2014. Moreover, when comparing the working age groups and entire life span, a relative importance of the disease is reported in Table 1. The relative weight is expressed as a share between the PGLE expected for the working group and the PGLE during entire life span.

**Table 1:** Added years of life at birth and for working ages after eliminating causes of death by sex, 2012–2014

Cause of death	Potential gains of life expectancy (in years)				
	Age group			(%)	
Sex	at birth	25–44	45–64	25–44/at birth	45–64/at birth
*Chronic Ischemic Heart Disease (I25)*					
Total population	3.55	0.0013	0.0377	0.04	1.06
Males	2.93	0.0019	0.0602	0.07	2.05
Females	3.97	0.0005	0.0148	0.01	0.37
*Cerebrovascular Diseases (I60-I69)*					
Total population	1.11	0.0018	0.0273	0.16	2.47
Males	1.03	0.0021	0.0394	0.21	3.81
Females	1.12	0.0015	0.0150	0.13	1.34
*Acute Coronary Syndrome (I20-I22)*					
Total population	0.70	0.0011	0.0340	0.16	4.89
Males	0.85	0.0017	0.0561	0.20	6.61
Females	0.49	0.0005	0.0116	0.11	2.36
*Hypertensive Diseases, exc. Secondary (I10-I13)*					
Total population	0.18	0.0004	0.0048	0.24	2.65
Males	0.16	0.0008	0.0063	0.50	3.93
Females	0.19	0.0001	0.0033	0.03	1.71
*Atherosclerosis, Aortic Aneurysm and Dissection (I70-I72)*					
Total population	0.16	0.0005	0.0050	0.30	3.14
Males	0.15	0.0009	0.0073	0.59	4.76
Females	0.15	0.0000	0.0026	0.01	1.69
*Diabetes Mellitus (E10-E14)*					
Total population	0.14	0.0006	0.0047	0.45	3.48
Males	0.12	0.0004	0.0060	0.35	5.04
Females	0.15	0.0008	0.0034	0.55	2.32

The highest values of PGLE at birth among all Slovak population are stated from the elimination of chronic ischemic heart disease (3.55 years), followed by cerebrovascular diseases (1.11 years), acute coronary syndrome (0.7 years), hypertensive diseases (0.18 years), atherosclerosis (0.16 years), and diabetes mellitus (0.14 years). The impacts of deaths from chronic ischemic heart disease, cerebrovascular diseases, hypertensive diseases and diabetes mellitus on the length of life are higher for females than those for males. On the other hand, males would live longer than females, if mortality of acute coronary syndrome was eliminated. In the case of atherosclerosis elimination, the PGLEs at birth of both sexes are the same. It is worth noting that women are more burdened of chronic heart disease than men, while the burden of acute heart disease is higher for men compared with women. Thus, after elimination of chronic ischemic heart disease deaths, a woman at birth could be expected to live 3.97 years longer than the actual life expectancy at birth for females. While a man at birth could be expected to live 2.93 years longer than the actual life expectancy at birth for males.

When working ages are compared, the impacts of deaths from the each of diseases are higher for males than females, except the impact of diabetes mellitus in the age group 25–44. Consideration population of young adults (25–44 years), its labour force potential is most threatened by mortality of cerebrovascular diseases. By eliminating the deaths from cerebrovascular diseases, the duration of remaining working life of young adults would be prolonged by 0.0018 years of life. As for population of adults (45–64 years), its labour force potential is most threatened by chronic ischemic heart disease as well as acute coronary syndrome.

As one would notice, in spite of the PGLE of chronic ischemic heart disease (0.0602 years) for males of adults is higher than the PGLE of acute coronary syndrome (0.0561 years), the PGLE for working age 45–64 by elimination of chronic ischemic heart disease represents only 2.05% of the PGLE at birth, as compared with 6.61% for acute coronary syndrome. The same finding is seen when the PGLE at birth of chronic ischemic heart disease (2.93 years) is higher than the PGLE at birth of acute coronary syndrome (0.85 years) for males, however, the PGLE for working age 45–64 by elimination of chronic ischemic heart disease represents less contribution than acute coronary syndrome. Similar results are observed for people aged 25–44 years by eliminating two mentioned causes of death, although, a highest relative weight accounting for 0.59% is found for males by eliminating atherosclerosis.

## Discussion

The aim of this analysis was to quantify the impact of deaths from cardiovascular diseases and diabetes mellitus on life expectancy of the Slovak population in the working age groups by sex from 1996 to 2014 and to reveal the relative importance of causes of deaths when comparing the entire life span and working life-time.

According to results, the main problem, that should be primarily solved by health policy planners, relates to the deaths from chronic ischemic heart disease in the working age (45–64 years) for males as well as females. The finding results from the long-term observation during the last two decades. It is one of the ways by which the life expectancy could be faster prolonged and also the economic production could be higher. When consideration past three examined years in terms of the PGLE at birth, life expectancy could be most extended by elimination of deaths from chronic ischemic heart disease for females, and acute coronary syndrome for males. In this case, we have to take into account that the PGLE at birth considers the entire life span, hence, additionally the people aged 65 over. In terms of the PGLE for both sexes of working group (25–44 years), the labour force potential is most threatened by deaths from cerebrovascular diseases, while deaths from chronic ischemic heart disease for population of working age (45–64 years).

In addition, we revealed that a relative importance of some diseases can change when comparing the entire with working time life. The relative importance of acute coronary syndrome for males aged 45–64 years increased when the share of PGLE (45–64 years) on PGLE at birth was calculated. Our findings suggest that it is very important to determinate whether the PGLEs at birth or working life time are considered, and then decide about the most burdens of diseases. Obviously, the years gained during the two sets of working life do not have the same weight in terms of economic costs of diseases.

One of the reasons for the high differences in the CVD between males and females, supported by previous studies [22–24], may be the fact that women in reproductive age are protected by hormones, thus have a lower prevalence of CVD. On the other hand, women in menopause are at a higher risk than men.

Nevertheless, there are any specific CVD prevention or treatment procedures that would take into account gender differences in Slovakia. Henceforth, the health policy makers should focus on eliminating risk factors (e.g. LDL cholesterol, dietary habits, physical activity, smoking) and compensation of already existing diseases (hypertension, diabetes mellitus – type 2, dyslipidemia atherogenic, metabolic syndrome).

Unfortunately, a national program of CVD prevention as well as treatment guidelines do not exist in Slovakia. Concurrently, the general practitioners are ageing and they do not have a motivation for education, that leads to the incorrect treating patients. This interpretation is consistent with the DYSIS study by Pella et al. (not published yet), who found that only a fifth patients had met the target for LDL-C and achieved optimal levels of other screened lipids.

Our surprising findings, that the labour force potential of working group (25–44 years) is most threatened by deaths from cerebrovascular diseases and working group (45–64 years) by the deaths from chronic ischemic heart disease, can be explained by gradually shifting morbidity into the younger age groups of the Slovak population [25].

Our result also suggests that women aged 25–44 are more threatened by deaths from diabetes mellitus than men aged 25–44. This finding appears to reflect the fact that women in younger age suffer from gestational diabetes or subsequent development of postpartum diabetes, obesity, higher weight after a childbirth, metabolic syndrome, insulin resistance. Our statements are in agreement with the studies by Martinka et al. [7], Šulcová et al. [26], Wild et al. [5].

To reduce the prevalence of diabetes mellitus can help these possible strategies: reducing preventable risk factors, such as obesity; screening; improve the diagnosis and monitoring of blood glucose diabetics; improve the treatment of vascular complications, including diseases of the kidney, retinopathy, diabetic legs and other neuropathies; and improved monitoring of cardiovascular risk.

Additionally, the potential gain expected for males working age 25–44 by elimination deaths from atherosclerosis represents 0,59% of the expected gain for the entire life span, while the lower shares are observed in other diseases. As for men in working age 45–64, the highest share was found in acute coronary syndrome. These results confirm that atherosclerosis is the main risk factor associated with an increased risk of acute coronary syndrome, and it is a warning signal indicating the expected increase in deaths from acute coronary syndrome.

### Limitations

While the potential gains in life expectancy seems to be a beneficial indicator for measuring the burden of diseases, it has some limitations. According to this indicator, life expectancy is extended by elimination of deaths. However, life expectancy is also affected by many other factors, e.g. quality of health care, life style, crime rates, economy, state military status, environment, and others. Moreover, total elimination of a certain cause of death is not likely, on the other hand, it provides the real strength to the other competing risks of causes of death. It may seem that the extension of life expectancy through eliminating deaths from a disease in the working age groups by several days is negligible and unimportant. However, we have mentioned in introduction section that life expectancy prolonged by 4 years from 1996 to 2014, that is quite a long duration, therefore an extension of life expectancy only by a few days means a substantial progress.

## Conclusions

Based on the observed law values of the potential gains in life expectancy for the working population, we can assume that the substantial part of the burden of examined diseases refers to the people aged 65 over. However, from the term of sustainability of health system financing and economic growth, it is desirable to highlight the relative importance of causes of death during the working life of Slovak population.

In spite of limitations, the PGLE is an innovative indicator for measurement the competing risks of causes of death expressing the value to which the lifetime could be extended. Comprehensive analysis is a basis for creating the strategic solutions in prevention, diagnostics and treatment guidelines in the Slovak Republic.

## Abbreviations

CVD: Cardiovascular diseases
ICD-10: The Tenth Revision of the International Classification of Disease
PGLEs: Potential gains in life expectancy
